# A Partly Fermented Infant Formula with Postbiotics Including 3′-GL, Specific Oligosaccharides, 2′-FL, and Milk Fat Supports Adequate Growth, Is Safe and Well-Tolerated in Healthy Term Infants: A Double-Blind, Randomised, Controlled, Multi-Country Trial

**DOI:** 10.3390/nu12113560

**Published:** 2020-11-20

**Authors:** Yvan Vandenplas, Virginie de Halleux, Małgorzata Arciszewska, Piotr Lach, Valeriy Pokhylko, Viktoriia Klymenko, Stefanie Schoen, Marieke Abrahamse-Berkeveld, Kelly A Mulder, Rocio Porcel Rubio

**Affiliations:** 1 KidZ Health Castle, UZ Brussel, Vrije Universiteit Brussel, 1090 Jette, Belgium; 2 Centre Hospitalier Universitaire of Liège (CHU), Centre Hospitalier Régional of Liège (CHR), 4000 Liège, Belgium; vdehalleux@chuliege.be; 3 Poliklinika Ginekologiczno-Położnicza Sp. z o.o. Sp. k., 15-435 Bialystok, Poland; marta@arciszewska.eu; 4 Centrum Medyczne Promed, 31-411 Kraków, Poland; alach@cmpromed.pl; 5 Ukrainian Medical Stomatological Academy, Poltava Regional Children Clinical Hospital, 36011 Poltava, Ukraine; v.i.pokhylko@gmail.com; 6 Kharkiv National Medical University, City Children’s Clinical Hospital No. 19, 61051 Kharkiv, Ukraine; klymenkoviktoriia@gmail.com; 7 Danone Nutricia Research, 3584 CT Utrecht, The Netherlands; stefanie.schoen@danone.com (S.S.); marieke.abrahamse@danone.com (M.A.-B.); kelly.mulder@nutricia.com (K.A.M.); 8 Hospital Quironsalud, 08023 Barcelona, Spain; rocio.porcel@quironsalud.es

**Keywords:** safety, tolerance, infant growth, prebiotic, postbiotic, 2′-linked fucosyllactose (2′-FL), 3-galactosyllactose (3′-GL)

## Abstract

This study investigated growth, safety, and tolerance in healthy infants consuming a partly fermented infant formula (IF) with postbiotics, 2′-linked fucosyllactose (2′-FL), a specific prebiotic mixture of short-chain galacto-oligosaccharides (scGOS) and long-chain fructo-oligosaccharides (lcFOS), and milk fat. This double-blind, controlled trial randomised 215 fully IF-fed infants ≤ 14 days of age to either: Test Group (IF) containing 26% fermented formula with postbiotics derived from Lactofidus fermentation process (including 3′-Galactosyllactose; 3′-GL), 0.8 g/100 mL scGOS/lcFOS (9:1), 0.1 g/100 mL 2′-FL, and milk fat), or Control group (IF with 0.8 g/100 mL scGOS/lcFOS (9:1)) until 17 weeks of age. Fully breastfed infants were included as a reference. Anthropometric measures, gastrointestinal symptoms, and safety were assessed monthly. Equivalence in weight gain (primary outcome) between the Test and Control groups was confirmed (difference in means −0.08 g/day; 90% CI (−1.47;1.31)) with estimated mean weight gain (SE) of 31.00 (0.59) g/day and 31.08 (0.60) g/day, respectively, (PP population, *n* = 196). Equivalence in length and head circumference gain between the randomised groups was also confirmed. No statistically significant differences were observed in adverse events or gastrointestinal tolerance between randomised IF groups. A partly fermented IF with postbiotics, specific oligosaccharides, 2′-FL, and milk fat supports adequate infant growth and is safe and well-tolerated in healthy term infants.

## 1. Introduction

Nutrition in early life has a fundamental impact on the growth and development of infants, especially during the first 1000 days. The unique composition of human milk is ideal to support this critical developmental period. Human milk feeding has been linked among other benefits to growth [1], reduced infections and illnesses [2], and brain development [3]. Although the specific nutritional and bioactive compounds in human milk conferring these health benefits are not yet fully understood, research is increasingly focusing on the potential importance of oligosaccharides present in human milk (HMOs).

Human milk contains over 200 different HMOs [4,5]. Their action is synergetic and fulfils complex roles in the development of the infant’s gut microbiome, immune system, and brain [6,7,8]. While the same five monosaccharides provide the building blocks of all HMOs, the structure and functionality differ. The composition of HMOs in human milk is known to vary across lactating women [6,9]. The most prevalent HMO among the majority of women is 2′-Fucosyllactose (2′-FL), and it is thought to have a role in the gut and immune system of the developing infant [10,11]. The concentrations of individual HMOs in human milk differ during lactation and decrease over time [5,12]. For example, the amount of 2′-FL ranges from 0 to 4.5 g/L in early lactation [13], whereas 3′-Galactosyllactose (3′-GL) has been reported as high as 0.08 g/L in colostrum and ranges from 0.005 to 0.039 g/L over the first 21 days [14,15]. However, these values are based on a small number of samples and further work is required to determine the genetic, dietary, and other environmental factors that impact the natural variability in concentration of HMOs in mother’s milk.

When a mother is not able or chooses not to breastfeed, providing infant formula (IF) is the most suitable alternative, aiming to provide nutritional and functional properties as close as possible to those of human milk. Clinical studies have shown that supplementation of IF with a specific prebiotic mixture of short-chain galacto-oligosaccharides (scGOS) and long-chain fructo-oligosaccharides (lcFOS) (9:1), a ratio mimicking the diversity of oligosaccharides present in human milk, leads to a more favourable gut microbiota composition and activity, closer to that observed in breastfed infants [16,17]. Moreover, scGOS and lcFOS in IF has been associated with a lower number of infections, fever episodes, and antibiotic prescriptions [18]. Likewise, the addition of HMOS including 2′-FL to IF has been suggested to result in lower inflammatory markers [19] and fewer parental-reported bronchitis and respiratory tract infections [20].

In addition to human milk, the HMO 3′-GL has also been identified in fermented IF as a by-product of the fermentation process, and is also known as a postbiotic [15]. Although an official definition is still pending, the term postbiotic describes the compounds produced by microorganisms and released from food components or microbial constituents, including non-viable cells that, when administered in adequate amounts, promote health and well-being [21]. While the role and mechanisms of these compounds is not yet fully understood, it is possible that they may contribute to the gastrointestinal benefits associated with fermented IF [22,23]. Previously, providing the combination of postbiotics (including 3′-GL) and scGOS/lcFOS in an IF was shown to result in softer stools and a more favourable microbiota composition and activity closer to that of breastfed infants [24,25,26].

In a continuous effort to improve IF aiming to support the infant during a critical period of development, a novel formula with specific oligosaccharides was developed. This study investigated growth, safety, and tolerance of this novel IF containing postbiotics (including 3′-GL) from the Lactofidus fermentation process, a specific prebiotic mixture (scGOS/lcFOS), 2′-FL, and milk fat.

## 2. Materials and Methods

### 2.1. Participants

This multi-centre study included infants recruited from study centres in Belgium (three centres: KidZ Health Castle, UZ Brussel; CHU-CHR Liège; AZ Delta), Hungary (three centres: Gyerkőc-med Bt; ClinExpert Healthcentre; Bugpat Hospital), Poland (seven centres: Poliklinika Ginekologiczno- Położnicza Sp. z o.o. Sp. k.; NZLA Michalkowice Jarosz i partnerzy Spolka Lekarska; CM Promed; Specjalistyczna Poradnia Medyczna Przylądek Zdrowia; Centrum Medyczne Plejady; Gdańskie Centrum Zdrowia; Korczowski Bartosz, Gabinet Lekarski), Spain (seven centres: Department of Paediatrics at Reina Sofía Children’s Hospital; A Coruña University Hospital; Hospital Universitario San Cecilio; Hospital Quironsalud, Servicio de Pediatría. CCEE; Hospital General Universitari D’elx; EBA Centelles; CAP Peralada), and Ukraine (four centres: Lviv National Medical University, Department of Pediatrics #1, based on Municipal City Children Hospital; Higher state educational institution of Ukraine “Ukrainian medical stomatological academy”; Vinnytsya Regional Children’s Hospital; Municipal Nonprofit Enterprise “City Children’s Clinical Hospital № 19”). Healthy infants ≤ 14 days of age were enrolled in this study, with fully IF fed infants randomised to the Test or Control IF. Fully breastfed infants were included as a reference group. The term fully breastfed was derived from the WHO definition for predominant breastfeeding (allowing small amounts of water, vitamins, and medicines) with the exception that we did not allow donor milk. Fully IF fed infants were also allowed small amounts of water, vitamins, and medicines. Additional inclusion criteria were gestational age between ≥37 weeks + 0 days and ≤41 weeks + 6 days, birth weight within 10th–90th percentile for gestational age and sex (according to intergrowth standards [27] or local growth charts), head circumference at birth within ±2 SD of WHO Growth Standards [28] for age and sex, and without any current or congenital illness/disease that could interfere with the study or its parameters. Infants who required a special diet, had a suspected allergy or intolerance to cows’ milk, lactose, or soy, or had participated in a previous or current clinical study involving another product were not eligible for participation. For the lactating mothers of infants included in the Breastfed reference group, additional inclusion criteria were defined: no significant medical conditions which might interfere with the study or its outcome, and no participation in any study involving interventional products.

### 2.2. Study Design

This was a double-blind, randomised, controlled, multi-country, two-arm parallel group growth equivalence trial. This trial was registered in ClinicalTrials.gov (identifier: NCT03476889). Infants of parents who autonomously decided to fully formula feed were block randomised (block size of 4) on a 1:1 basis, stratified by study site and sex, to receive the Test IF or Control IF until 17 weeks of age. The randomisation sequence was generated using the PLAN procedure of SAS statistical software by a methodologist with no further involvement in the conduct of the study, and the IF packed in tins was labelled with unique codes by a clinical studies supplies manager, also with no further involvement in study conduct. The randomisation sequence was uploaded in a central interactive web-response system (IWRS) which was accessed by the investigator to obtain the randomly assigned unique code upon enrolment of an infant to the study. The unique product code corresponded to either the Test or Control IF which were stored at the study site. The tins with the unique product code were dispensed to the parent of the infant during study visits. Parents and study staff were all blinded to the IF assignment. The breastfed infants enrolled as a reference completed the same study procedures as the randomised, IF fed infants.

This study was conducted in compliance with the principles of the Declaration of Helsinki and all procedures were reviewed and approved by the local Ethical Review Boards of participating centres. Prior to screening, written informed consent was obtained for all infants from parent(s)/legal guardians (referred to hereafter as parents).

### 2.3. Study Products

The Test IF and Control IF were both nutritionally complete cows’ milk based IF (Directive 2006/141/EC), containing similar amounts of intact protein (1.3 g/100 mL), lipids (3.4 g/100 mL), and carbohydrate (7.3 g/100 mL), and were produced in accordance with good manufacturing practices (ISO 22000) (Table 1). The products were provided in identical 400 g tins. The Test IF contained 26% fermented formula with postbiotics derived from the Lactofidus fermentation process (including 3′-GL). The unique Lactofidus fermentation process involves the addition of *Bifidobacterium breve* C50 and *Streptococcus thermophiles* 065 to IF and is followed by a mild heat treatment. Additionally, 0.8 g/100 mL scGOS/lcFOS (9:1), and 0.1 g/100 mL 2′-FL was added to the Test IF which also contained anhydrous milk fat (49.8% of total fat). The Control IF was a commercially available standard IF containing the oligosaccharides scGOS/lcFOS (0.8 g/100 mL; 9:1), but no 2′-FL, postbiotics, or milk fat. The infants were to be fed ad libitum with only the study product during the entire intervention period. Infants in the reference arm were to be fully breastfed fed ad libitum.

### 2.4. Measurements

The primary parameter in this study was weight gain (g/day) from baseline until 17 weeks of age. Secondary parameters included the gain (cm/day) in length and head circumference, the corresponding z-scores of anthropometric measurements, parent-reported gastrointestinal (GI) tolerance (regurgitation, vomiting, stool characteristics), and safety outcomes (investigator-reported adverse events). The study visits were performed at the infants age of ≤14 days of age (baseline/V1), 4 weeks (V2), 8 weeks (V3), 12 weeks (V4), and 17 weeks (V5). During the baseline visit, written informed consent was obtained from the parent(s) and eligibility was checked. Fully formula fed infants were randomised to one of the two study products, and fully breastfed infants were enrolled in the reference group. For all infants, demographics, family characteristics, and relevant medical history were collected by interview. At all study visits, anthropometrics were measured. Infant (naked) body weight was measured to the nearest gram using a calibrated electronic infant weighing scale (KernMBC 20K10M, Kern & Sohn GmbH, Germany). Infant length was measured to the nearest 0.1 cm using a length board (KernMBC A01, Kern & Sohn GmbH, Germany). Head circumference was measured with a non-stretchable measuring tape (seca 212, seca, Germany). All anthropometrics were measured twice, and if a substantial difference between measurements occurred (>50 g for weight and >5 mm for length and head circumference) a third measurement would be taken. The two closest measurements were averaged for the statistical analysis.

A seven-day paper diary was used to collect information about GI symptoms, stool characteristics, and daily IF intake during the week after the baseline visit and the week prior to each subsequent study visit. The number of regurgitation (spitting up; return of the milk into the mouth without force) and vomiting (return of the milk into the mouth with force) occurrences were recorded. Stool consistency was scored by the parents for each stool passed using the Amsterdam Stool Scale (watery, soft, formed, hard) [29]. For formula fed infants, study product intake was recorded as the volume prepared (including water and scoops of powdered formula) and the volume left over. All diaries were reviewed for completion and plausibility by the investigator at each visit. In addition, investigators also reported all adverse events (AE) including severity, relation to study product, and actions taken, as well as any concomitant medication.

### 2.5. Statistical Analysis

The primary objective of this study was to test the equivalence in daily weight gain from baseline until 17 weeks of age in infants receiving the Test IF compared to infants receiving the Control IF. To demonstrate equivalence in daily mean weight gain between the randomised groups, the required sample size of 192 randomised infants (96 per group) was calculated with the two-one sided tests (α = 0.05; power = 0.80; drop-out/non-compliance rate = 25%). The a priori assumptions included a margin of equivalence of ±3 g/day [30] and an equal within-group standard deviation (SD) of 6.1 g in weight gain in both the Test and Control IF groups. The estimated difference in mean daily weight gain between the two IF groups was assumed to be zero. Two interim analyses were performed; one to evaluate early safety outcomes and a second to re-evaluate sample size. The independent Data Monitoring Committee concluded that the study could continue without modification after both interim analyses.

The per protocol (PP) population was used as the primary analysis dataset for all analyses including weight, length, head circumference, and WHO z-scores. The PP population was determined at visit level, meaning that infants data was included in the PP analyses until a major protocol violation occurred, including stopping study product intake and/or start of commercial IF or complementary food. The PP population included infants who met all eligibility criteria, had at least one post-baseline weight measurement, and consumed only the study product (or were fully breastfed for infants in the reference group). Infant weight gain was modelled with a Parametric Growth Curve (PGC) mixed model with a quadratic function of time. The stratification factors of infant sex and study site, and additionally birth weight were included as covariates. Two sensitivity analyses were performed, one adding gestational age as a covariate and a second excluding influential subjects. Equivalence of infant length and head circumference gain were analysed using the same statistical model but applying an equivalence margin of 0.5 SD. As one of the secondary objectives, an equivalence analysis of gain in anthropometrical parameters between the Test and Breastfed reference group was performed. For the daily weight and length gain equivalence analysis of the Test group and Breastfed reference group, maternal BMI was also added as a covariate, and maternal education level was added as an additional covariate for length gain. Anthropometric z-scores were calculated using a macro provided by the World Health Organisation (WHO) [28] and were analysed using a Mixed Model Repeated Measures (MMRM) using visit as a categorical variable, with the stratification factors infants’ sex and study site and additionally birth weight as covariates. In addition, maternal BMI was included as a covariate in a three-arm model when the analysis included the Breastfed reference group.

The analysis of safety and tolerance, comprising of the comparison of AEs and parent-reported GI tolerance of the IF groups, was performed using the All Subjects Treated (AST) dataset, which includes all infants with at least one feeding of the study product. The parent-reported GI tolerance and study product intake parameters were calculated from the parent-report diaries for all infants with at least three out of seven days of data completed per diary. GI tolerance (regurgitation, vomiting, stool characteristics) were analysed by visit and include for regurgitation: (i) occurrence of regurgitation at least once on one day; (ii) occurrence of frequent regurgitation (≥ 3 times on 40% of diary days); and for vomiting (i) occurrence of vomiting on at least one day; (ii) occurrence of vomiting on at least 40% of diary days. A cut off of 40% of diary days was chosen as this roughly reflects half of the week with a specific occurrence on 3 out of 7 diary days. Stool characteristics include: (i) mean stool frequency (number of stools/day); (ii) stool consistency was evaluated for each infant and is presented as the percentage of infants per category (watery, soft, formed, hard) based on their mean stool consistency score over all diary days within the visit. The occurrence of infrequent hard stools (two or fewer defecations per week which have a hard consistency) and frequent watery stools (passage of three or more watery stools in a day) were also calculated from the stool consistency and frequency data per visit.

Study product intake parameters were analysed using the PP and the All Subjects Randomised (ASR) population. Study product intake (mL/day) is presented per visit as the infants’ mean daily intake (volume of product prepared minus left over) over the recorded days, if at least three days in the diary had no missing data. Diaries with less than three completed days were not included in the analysis and recorded as missing data. Additionally, infants’ mean daily IF intake by body weight (mL/kg) was calculated for each visit.

Continuous parameters were analysed with the t-test or Wilcoxon rank sum test and the Miettinen and Nurminen approach was used for comparison of binary parameters, with a restriction for the adverse events evaluation having-p-values generated only when the event incidence was at least four in either group. Estimates of the risk difference in the percentage of infants between the Test and Control groups were calculated along with corresponding Miettinen and Nurminen 95% confidence intervals. Categorical parameters were analysed with a chi-square test. Decisions to assign infants to the individual datasets (AST, PP) were made on fully blinded data during a data review meeting prior to the analyses. All statistical analyses were conducted according to a pre-defined statistical analysis plan using SAS^®^ (SAS version 9.4_TS1M3 or higher in SAS Life Science Analytics Framework version 4.7.3 or higher) for LIN X64, SAS Institute Inc., Cary, NC, USA.

## 3. Results

### 3.1. Subject Characteristics

**Figure 1:**
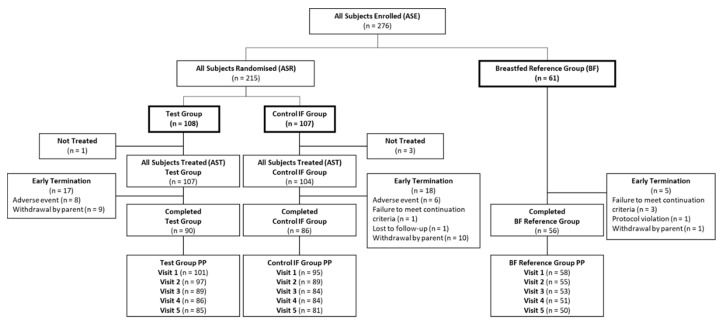
Flow diagram of participants through the study from enrolment to study completion for the All Subjects Treated, Completed, and Per Protocol populations. Completed refers to infants who remained in the study for the entire intervention period without early termination. The per protocol population was evaluated on the visit level and included all infants who met all inclusion criteria, including having at least one post-baseline weight measurement, and consumed only the study product during the intervention (i.e., no commercial infant formula, no complementary/weaning foods) for infants in the randomised groups, or were fully Breastfed for infants in the reference group.

Between June 2018 and April 2019, a total of 276 infants were enrolled in this study of which 215 infants were randomised to the Test or Control IF and 61 infants were enrolled in the Breastfed reference group (Figure 1). Due to fast enrolment towards the end of the recruitment period, 23 additional formula fed infants and 1 additional breastfed infant were enrolled than initially planned. The entire study was completed for 90 infants in the Test, 86 in the Control, and 56 in the Breastfed reference groups. The dropout rate was similar between the Test (16%) and Control (17%) groups; 85 and 81 infants in the Test and Control groups, respectively, completed the last visit (V5) according to the protocol. For the Breastfed reference group, the dropout rate was 8% and 50 infants completed V5 according to the protocol (Figure 1).

No relevant differences were observed in the demographic characteristics of the randomised groups, with a median (Q1–Q3) of 10 (5–12) days of age at baseline, 52.6% female, and 50.5% were born by caesarean section among all infants randomised (Table 2). Over half (54.6%) of the IF-fed infants were enrolled in Poland, 16.3% in Spain, 14.3% in Ukraine, 10.7% in Belgium, and 4.1% in Hungary. Evaluation of demographics of the Breastfed reference group indicated some differences compared to the IF-fed infants. The most substantial differences included a lower rate (34.5%) of infants born by caesarean section and a higher rate of breastfed infant mothers who completed a tertiary education (79.3%) compared to the randomised infant mothers (36.7%).

### 3.2. Study Product Intake

During the intervention period, completion of the feeding diaries prior to the visits ranged between 90% and 98% of all randomised infants enrolled in the study. No statistically significant differences in IF intake were observed between the Test and Control groups at any timepoint. The mean (SD) daily volume of formula consumed increased from 598 (130) mL/day in the week post-baseline to 1007 (225) mL/day at week 17 in the Test group and from 626 (147) mL/day to 1022 (220) mL/day in the Control group (Table 3). Similarly, there were no statistically significant differences in intake per kg body weight (mL/kg/day) between the groups (Table 3).

### 3.3. Growth Outcomes

As the primary outcome, equivalence in daily weight gain (g/day) between the Test group and the Control group from baseline to 17 weeks of age was demonstrated. The difference in the estimated means (SE) of daily weight gain between the two groups was −0.08 (0.84) g/day, 90% CI −1.47, 1.31. The estimated mean (SE) daily weight gain was 31.0 (0.59) g/day in the Test group and 31.08 (0.60) g/day in the Control group. The average total and daily weight gain for the entire intervention period are shown in Table 4 and the absolute weight at the specific visits are shown in Supplementary Table S1.

Equivalence was also confirmed for both length and head circumference gain between the Test and Control groups (data not shown). The estimated means for total and daily length and head circumference gain for the entire intervention period are shown in Table 4. The absolute values at the specific visits are shown in Supplementary Table S1.

**Figure 2:**
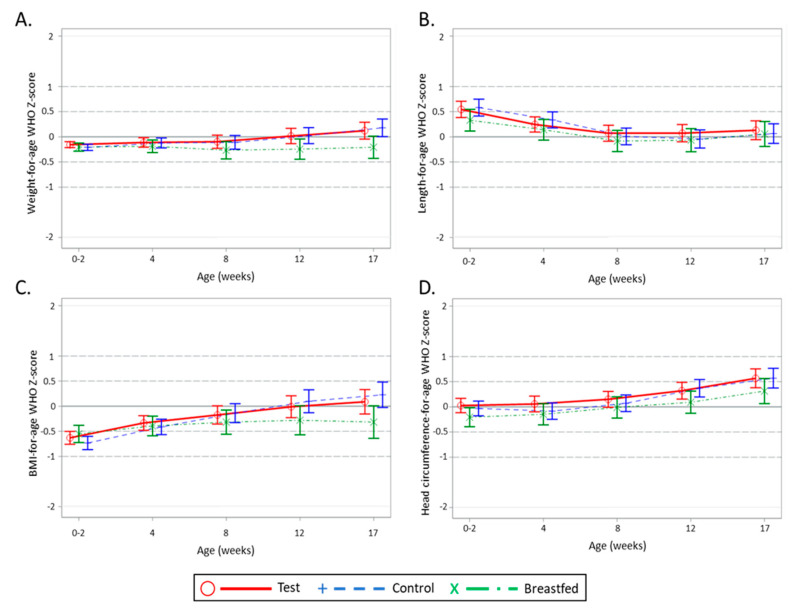
Estimated mean (± 95% CI) WHO Growth Standard z-scores weight-for-age (**A**), length-for-age (**B**), BMI-for-age (**C**), and head circumference-for-age (**D**) per visit for the Test, Control, and Breastfed reference groups. Per protocol population and Breastfed reference group.

The mean (SE) daily weight gain of the Breastfed reference group of 28.3 (0.79) g/day appeared slightly lower than the Test group and was not equivalent, with an estimated mean (SE) difference of 2.65 (0.99) g/day, 90% CI (1.01, 4.29). Equivalence was confirmed for both length and head circumference gain between the Test group and Breastfed reference group (data not shown). Notably, in comparison with the WHO growth standards, the estimated z-scores for weight-for-age, length-for-age, BMI-for-age, and head circumference-for-age of the randomised groups as well as the Breastfed reference group were all within +/−1 SD bandwidth, indicative for adequate infant growth (Figure 2).

### 3.4. Parent-Reported GI Tolerance

The majority of randomised infants (>75%) experienced at least one day with regurgitation at visits under 12 weeks of age, and 67.4% at 12 weeks (V4) and 65.0% at 17 weeks (V5) of age. The occurrence of regurgitation was slightly higher in the Breastfed reference group decreasing from 86.4% of infants at V1 to 73.2% at V5. The highest incidence of frequent regurgitation (≥3 occurrences per day on at least 40% of diary days) was reported at 8 weeks of age (V3) with 23.4% of infants in the Test group, 25.8% of infants in the Control group (*p* = 0.80), and 45.8% of infants in the Breastfed reference group.

Vomiting was not reported for >70% of the randomised infants during the entire intervention period with no statistically significant differences between groups at any timepoint (data not shown). In line with these observations, the occurrence of vomiting was also low among infants in the Breastfed reference group, with no vomiting reported for >70% of Breastfed infants. The occurrence of vomiting on 40% of diary days was 11.8% for the randomised infants at V1 and 4.7% at V5, and for the Breastfed reference group was 13.8% and 3.7% of infants at V1 and V5, respectively (data not shown).

During the intervention period, the median daily stool frequency decreased from 2.6 to 1.4 in the Test group and 2.6–1.3 in the Control group from V1 to V5, respectively (*p* > 0.05) (Supplementary Figure S1). Notably, variability in stool frequency was high at V1 with Q1–Q3 of 1.7–4.0, and at V5 was 1.0–1.9 among all randomised infants. The median stool frequency in the Breastfed reference group was higher with a median (Q1–Q3) of 5.1 (3.6–6.3) at V1 and 1.7 (0.6–3.4) at V5. 

**Figure 3:**
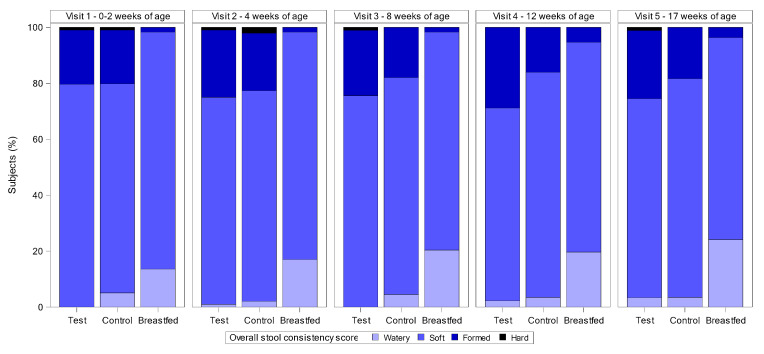
Percentage of infants per stool consistency category based on their mean stool consistency visit per visit. All subjects treated population and Breastfed Reference. Chi-square test was used for comparison between the Test and Control groups (V1: *p* = 0.147; V2: *p* = 0.803; V3: *p* = 0.119; V4: *p* = 0.120; V5: *p* = 0.569). Number of subjects per group for Test, Control, and Breastfed group, respectively—V1: *n* = 103/99/59; V2: *n* = 100/97/59; V3: *n* = 94/89/59; V4: *n* = 90/87/56; V5: *n* = 90/87/56.

Among infants in the randomised groups, the percentage of infants with a mean score of either watery or hard stools was very low at all timepoints (Figure 3). Less than 5% of infants had a mean score of watery, and <2% of infants had a mean score of hard with no statistically significant differences between the two randomised groups at any timepoint (*p* > 0.05). Watery stools appeared to be higher in the Breastfed reference group, with a mean score of watery for 13.6% of infants at V1 and 24.1% of infants at V5. No infants in the Breastfed reference group had a mean score of hard stools at any timepoint. Soft stool was the mean score among the randomised infants for > 70% of the infants at all timepoints and formed stools was the mean score for 19.3% of the infants at V1 and 21.5% of the infants at V5, and again there were no statistically significant differences between the test and control groups. For the Breastfed reference group, soft stool was also the most frequent mean score, ranging from 84.7% of infants at V1 to 72.2% of infants at V5. Formed stools were infrequent among the infants in the Breastfed reference group, with between 1.7 and 5.4% of infants having a mean score of formed stools during the study period (Figure 3).

Infrequent hard stools (two or fewer defecations per week, with a hard consistency) were not detected in any of the parent-reported diaries in the randomised groups and were recorded for two infants in the breastfed reference group at 17 weeks of age (V5). Frequent watery stools (passage of three or more stools with watery consistency) were present in a small percentage of the IF fed infants with no statistically significant differences between the randomised groups at any timepoint. The highest incidence of frequent watery stools was 7.4% of infants at V1 (0–2 weeks of age), and the lowest incidence was 2.7% at V3 (8 weeks of age). In the Breastfed reference group the incidence of frequent watery stools was present in 32.2% of infants at V1 and decreased to 21.4% of infants at V5.

### 3.5. Adverse Events

At least one AE was reported in 39.3% of infants in the Test group and 31.7% in the Control group with an estimated risk difference of 7.52% and a corresponding 95% CI of −5.42%, 20.22% (*p* = 0.255). For reference, an AE was reported for 24.6% of infants in the Breastfed reference group. The most common AEs occurred in the system organ class of gastrointestinal disorders which were reported in 20.6% of infants in the Test group, 16.3% in the Control group, and 9.8% in the Breastfed reference group (Table 5). Infections and infestations were reported in 15.9% of infants in the Test group, 16.3% in the Control group, and 13.1% in the Breastfed reference group (data not shown). In contrast to the parent-reported stool characteristics where no infrequent hard stools were noted in the randomised groups, investigators reported constipation for four (3.7%) infants in the Test group, three (2.9%) infants in the Control group, and one (1.6%) infant in the Breastfed reference group. There were no statistically significant differences between the randomised groups in the number of total or specific AEs.

A total of 11 serious AEs (SAEs) was reported in the randomised infants with seven events reported in six (5.6%) infants in the Test group and four events in four (3.8%) infants in the Control group (*p* > 0.05). From these SAEs six events in five (4.7%) infants in the Test group and three events in three (2.9%) infants in the Control group were in the System Organ Class of infection and infestation. The remaining two events were a case of infantile vomiting in the Control group and a case of Rhesus incompatibility in the Test group. No statistically significant differences in any SAEs were observed between the Test and Control groups. All serious adverse events were described by the investigator as not related or unlikely related to the study product. No SAEs were reported for the Breastfed reference group.

## 4. Discussion

The present manuscript reports on the first study to investigate infant growth, safety, and tolerance of a partly fermented IF with postbiotics (including 3′-GL), a specific prebiotic mixture (scGOS/lcFOS), 2′-FL, and milk fat. We demonstrated equivalence in daily weight, length, and head circumference gain up to 17 weeks of age between the Test and Control group. The Test and Control IF differed not only by the addition of prebiotics, 2′-FL, postbiotics, and milk fat, but also in concentrations of sn-2 palmitic acid, alpha-linolenic acid, ARA and DHA. The Test IF supported an adequate growth, was well-tolerated, and no safety concerns were revealed given the absence of clinically relevant differences in the number and type of investigator-reported (S)AEs.

The analysis of the primary objective was achieved by demonstrating the equivalence in daily weight gain from baseline to 17 weeks of age in infants receiving the Test compared to the Control IF. In addition, the mean (SE) values of daily weight gain 31.00 (0.59) g/day and 31.08 (0.60) g/day in the Test and Control group in the current study are in line with previously published data from other studies evaluating IF [24,25,31]. We have previously reported a mean (SD) daily weight gain of 28.3 (7.4) g/day and 30.1 (6.6) g/day for infants fed a partly fermented formula with scGOS and lcFOS or a non-fermented IF without scGOS and lcFOS until 17 weeks of age [24]. Another study included a non-fermented IF containing scGOS and lcFOS as a control product which was similar to the control in the present study and the daily weight gain was comparable with an estimated mean (SE) daily weight gain of 31.4 (0.5) g/day [31]. In addition, in an inferiority study, the mean (SE) daily weight gain of 29.8 (0.60) g/day up to 4 months of age of infants receiving an IF containing 2′-FL (1.0 g/L) and lacto-N-neotetraose (LNnT, 0.5 g/L) was not significantly different to the 30.2 (0.58) g/day of infants receiving a standard IF [20].

As one of the secondary objectives of this study, we compared the daily weight gain of the non-randomised Breastfed reference group and the Test group, which was lower in the breastfed infants compared to infants of the Test group and equivalence in weight gain between these groups was not demonstrated. We did, however, demonstrate equivalence in both length and head circumference gain between these groups during the intervention. The observed weight gain value of the breastfed infants in the current study is close to the range of values previously reported for breastfed infants (26.8–30.3 g/day up to four months of age) [24,31,32,33]. Given the high similarity in weight gain velocity between both IF groups, we did not evaluate the equivalence between the Control group and the Breastfed reference group. It has long been known that the growth patterns between breastfed and formula fed infants differ, with formula fed infants gaining more weight on average than breastfed infants in the first year of life [34,35,36], without particular differences in length or head circumference [36]. The key drivers of this difference in weight trajectory have not yet been identified. Apart from potential differences linked to factors present in the milk, more recent studies have suggested that mode of feeding may have an impact, with infants fed expressed breastmilk from a bottle gaining more weight than infants fed directly from the breast [37,38]. In addition, in our study the Breastfed reference group was not a randomised group and it is possible that we did not fully adjust for all differences between IF fed and breastfed infants. Although differences exist in the early weight gain pattern, both of the IF groups as well as the Breastfed reference group have a mean weight (as well as other growth parameters) close to the median of the WHO growth standard during the study period. We therefore conclude that the current study demonstrated adequate infant growth for both the IF groups and the Breastfed reference group.

Previous studies have shown that IFs containing scGOS/lcFOS, fermented formula, 2′-FL, or milk fat individually or in combination with other ingredients are well tolerated [19,20,24,25]. In line with these findings, the combination of these components in the Test IF of the current study was also well tolerated, reflected by the absence of statistically significant differences between the IF groups for regurgitation, vomiting, frequent watery stools, or infrequent hard stools at any timepoint. The number of infants presenting with regurgitation and infantile colic were slightly higher in the Test than in the Control group (5.6 vs. 1.0% and 7.5 vs. 3.8%, respectively), albeit these differences were not statistically significant (*p* > 0.05). Moreover, the prevalence of both regurgitation and infantile colic was much lower than those reported in literature, which are around 20% [39]. Hard stools and constipation have previously been attributed to IF feeding and suggested to be related to calcium soaps formed in the intestinal lumen [40]. The addition of sn-2 palmitic acid to IF has previously been shown to reduce the formation of insoluble soaps and result in softer stools [41]. In the current study, the Test IF contained milk fat and therefore approximately triple the amount of sn-2 palmitic acid compared to the Control IF containing only vegetable oils as the lipid source (Table 1). However, the combination of milk fat with partly fermented IF, 3′-GL, scGOS/lcFOS, and 2′-FL did not lead to additional stool softening, as no differences in the prevalence of watery stools were observed when compared to the Control IF. It should be noted, though, that the vast majority (>80%) of parent-reported stools in the IF groups of this study were scored as soft. As a reference, in the Breastfed group the majority was scored as soft or watery. Moreover, the absence of infrequent hard stools detected in the parent-reported diaries and low prevalence of investigator reported constipation among the IF fed infants (3.3%) is postulated to be due to the previously reported stool softening effect of scGOS/lcFOS [17,26].

No statistically significant or clinically relevant differences were observed in the number or type of adverse events between the randomised groups suggesting that no safety concerns were revealed for infants consuming the Test IF. In contrast to preclinical work showing that 2′FL can support the immune system to reduce infection and inflammation [42,43,44], the current study did not observe any statistically significantly differences in investigator-reported infections and infestations. Recently, a significantly lower incidence of parent-reported bronchitis was reported in infants following an intervention with an IF containing 2′-FL and LNnT compared to a standard IF without oligosaccharides [20]. However, the overall number of reported AEs in the study was higher than in our study, with between 84.1% and 90.8% of infants with a parent-reported adverse event [20] compared to 35.5% of the randomised infants in our study with an investigator-reported adverse event. Another study showed a lower incidence of investigator-reported infections and infestations in infants consuming an IF with lower 2′-FL (0.2 g/L) and higher GOS (2.2 g/L) (11%) compared to an IF with higher 2′-FL (1.0 g/L) and lower GOS (1.4 g/L) (38%) and an IF with 2.4 g/L GOS only (28%) (*p* < 0.05) [45]. Again this incidence of reported AEs is higher than the incidence of infections and infestations in our study which was approximately 16% for the randomised groups and 13.1% in the Breastfed reference group. However, it is possible that differences between studies are related to differences in how parents and investigators report adverse events. It is of importance to note that both IFs in our study contained the specific mixture of scGOS and lcFOS (9:1 ratio) which has also been shown to reduce infections in healthy infants [18,46]. Moreover, the current study was not designed to detect differences in the incidence of infection or to determine if the combination of postbiotics (including 3′GL), scGOS/lcFOS, and 2′-FL has a long-term impact on the developing immune system. The impact of the Test IF on inflammatory markers measured in stool, blood, and saliva, as well as faecal microbiota and metabolic parameters collected in this study, are currently under investigation.

Although a strength of this study was that it was a randomised, double-blind, controlled design capturing growth, safety, and parent-reported tolerance data for 276 infants, there are some limitations that should be considered. The Test IF assessed in this study combined several different components and it is therefore not possible to describe the impact of the individual ingredients on infant outcomes. The study was conducted at 24 sites in five European countries and therefore the results may not be transferrable to all regions.

In conclusion, a partly fermented IF with postbiotics (including 3′-GL), a specific prebiotic mixture of scGOS/lcFOS, 2′-FL and milk fat supports adequate infant growth and is safe and well-tolerated in healthy term infants. Future clinical studies are required to evaluate the potential (long-term) impact of this IF on the infants’ developing immune and gastrointestinal systems.

## Figures and Tables

**Table 1:** Composition of the infant formulas provided in this intervention study ^1^.

		Test IF Per 100 mL	Control IF Per 100 mL
Energy	Kcal	66	66
Carbohydrates	g	7.3	7.3
scGOS/lcFOS	g	0.8	0.8
2′-FL	g	0.1	0
3′-GL	mg	15	0
Protein	g	1.3	1.3
Whey	g	0.7	0.8
Casein	g	0.7	0.5
Fat	g	3.4	3.4
Vegetable oil	g	1.6	3.3
Dairy lipids	g	1.6	0.1
Saturates	g	1.7	1.5
Palmitic acid	mg	593	581
sn-2 Palmitic acid	mg	202	66.9
Monounsaturates	g	1.1	1.3
Polyunsaturates	g	0.6	0.6
Linoleic acid (LA)	mg	448	445
α-Linolenic acid (ALA)	mg	54.9	82
LA:ALA	ratio	8.15	5.40
Arachidonic acid	mg	16.5	11
Docosahexaenoic acid	mg	16.5	10

2′-FL, 2′-Fucosyllactose; 3′-GL, 3′-Galactosyllactose FOS, fructo-oligosaccharides; GOS, galacto-oligosaccharides; ^1^ Parents were instructed to feed their infants ad libitum.

**Table 2:** Infant and family characteristics per group ^1,2^.

	Statistic	Test (N = 101)	Control (N = 95)	Total IF (N = 196)	Breastfed (N = 58)
**Sex**					
Female	n (%)	54 (53.5)	49 (51.6)	103 (52.6)	31 (53.4)
Male	n (%)	47 (46.5)	46 (48.4)	93 (47.4)	27 (46.6)
**Country**					
Belgium	n (%)	11 (10.9)	10 (10.5)	21 (10.7)	4 (6.90)
Hungary	n (%)	4 (3.96)	4 (4.21)	8 (4.1)	9 (15.5)
Poland	n (%)	54 (53.5)	53 (55.8)	107 (54.6)	28 (48.3)
Spain	n (%)	20 (19.8)	12 (12.6)	32 (16.3)	10 (17.2)
Ukraine	n (%)	12 (11.9)	16 (16.8)	28 (14.3)	7 (12.1)
Age at Baseline (days) ^3^	Median (Q1–Q3)	10 (5–12)	10 (6–12)	10 (5–12)	11 (8–13)
Gestational Age (days) ^4^	Mean (SD)	39.4 (1.1)	39.3 (1.2)	39.3 (1.2)	39.3 (1.2)
**Mode of Delivery**					
Caesarean Section	n (%)	49 (48.5)	50 (52.6)	99 (50.5)	20 (34.5)
Vaginal	n (%)	52 (51.5)	45 (47.4)	97 (49.5)	38 (65.5)
Birth Weight (g)	Mean (SD)	3390 (368)	3317 (334)	3354 (353)	3346 (329)
Birth Length (cm)	Mean (SD)	53.1 (3.1)	52.7 (2.9)	52.9 (3.0)	52.8 (2.7)
Birth Head Circumference (cm)	Mean (SD)	34.7 (1.1)	34.4 (1.1)	34.5 (1.1)	34.5 (1.1)
Maternal Age (years)	Mean (SD)	30.7 (5.5)	30.5 (6.0)	30.6 (5.7)	32.3 (4.9)
**Maternal Education** ^5^					
Primary or Less	n (%)	10 (9.9)	11 (11.6)	21 (10.7)	2 (3.4)
Secondary	n (%)	57 (56.4)	46 (48.4)	103 (52.6)	10 (17.2)
Tertiary	n (%)	34 (33.7)	38 (40.0)	72 (36.7)	46 (79.3)
Maternal pre-pregnancy BMI (kg/m^2^)	Mean (SD)	23.8 (4.9)	24.4 (5.8)	24.1 (5.4)	22.4 (2.8)
Paternal BMI (kg/m^2^)	Mean (SD)	27.4 (4.0)	27.1 (3.8)	27.2 (3.9)	26.9 (3.8)

N = Number of subjects in the analysis population, ^1^ Per protocol population and Breastfed reference,^2^ No statistical comparison testing was conducted on the infant and family characteristics, ^3^ Age at baseline was calculated as: baseline date (V1)—birth date, ^4^ Gestational age is calculated as: gestational age in weeks + (number of days/7), ^5^ Primary education includes primary/elementary/grammar school, Secondary education includes secondary school/high school/trade school or equivalent, and Tertiary education includes college/university bachelor/university master/doctor degree.

**Table 3:** Daily infant formula intake per day and per kg body weight ^1,2^.

	mL/day					mL/kg/day				
	Test		Control			Test		Control		
	*n* (Missing)	Mean (SD)	*n* (Missing)	Mean (SD)	*p*-Value ^3^	*n* (Missing)	Mean (SD)	N (Missing)	Mean (SD)	*p*-Value ^3^
	All Subjects Randomised population									
Visit 1	98 (10)	598 (130)	98 (9)	626 (147)	0.237	98 (10)	176 (38.5)	98 (9)	187 (45.3)	0.091
Visit 2	96 (9)	758 (147)	95 (6)	774 (161)	0.981	95 (10)	178 (31.4)	92 (9)	186 (37.0)	0.326
Visit 3	91 (3)	851 (184)	88 (4)	881 (183)	0.412	91 (3)	164 (33.4)	88 (4)	172 (35.4)	0.236
Visit 4	90 (3)	939 (176)	85 (4)	950 (207)	0.993	89 (4)	157 (28.9)	85 (4)	159 (37.0)	0.878
Visit 5	89 (1)	1007 (225)	85 (2)	1022 (220)	0.533	89 (1)	148 (31.6)	85 (2)	150 (35.8)	0.718
	Per Protocol population									
Visit 1	93 (8)	602 (125)	94 (1)	624 (141)	0.275	93 (8)	176 (37.8)	94 (1)	187 (44.1)	0.084
Visit 2	92 (5)	762 (147)	87 (2)	767 (162)	0.557	92 (5)	179 (31.7)	87 (2)	184 (36.7)	0.559
Visit 3	87 (2)	861 (173)	83 (1)	878 (180)	0.596	87 (2)	165 (31.8)	83 (1)	171 (34.4)	0.318
Visit 4	85 (1)	941 (178)	80 (4)	944 (207)	0.818	85 (1)	155 (28.7)	80 (4)	158 (36.8)	0.939
Visit 5	84 (1)	1009 (227)	79 (2)	1025 (224)	0.518	84 (1)	147 (31.9)	79 (2)	151 (36.3)	0.425

^1^ All Subjects Randomised population, ^2^ Average daily consumed volume per visit was calculated if at least three diary days were filled out completely, including volume of water and number of scoops used and the volume of the left over reported for each bottle consumed in the day, ^3^ Mann-Whitney test for comparison between Test and Control Groups.

**Table 4:** Gain in weight, length, and head circumference from baseline to visit 5 (17 weeks of age) ^1,2^.

	Test (*n* = 101)		Control (N = 95)		Breastfed (*n* = 58)	
	Mean Estimate (SE)	95% CI	Mean Estimate (SE)	95% CI	Mean Estimate (SE)	95% CI
Weight gain						
g	3416 (64.5)	3289, 3543	3425 (66.6)	3293,3556	3109 (87.1)	2938, 3281
g/day	31.0 (0.59)	29.8, 32.2	31.1 (0.60)	29.9, 32.3	28.3 (0.79)	26.7, 29.9
Length gain						
cm	11.0 (0.2)	10.6, 11.4	10.8 (0.2)	10.4, 11.2	11.1 (0.3)	10.6, 11.6
cm/day	0.10 (0.0)	0.10, 0.10	0.10 (0.0)	0.09, 0.10	0.1 (0.0)	0.10, 0.11
Head circumference gain						
cm	6.54 (0.1)	6.31, 6.77	6.64 (0.12)	6.41, 6.88	6.41 (0.15)	6.12, 6.71
cm/day	0.06 (0.0)	0.06, 0.06	0.06 (0.0)	0.06, 0.06	0.06 (0.0)	0.06, 0.06

^1^ Per protocol population and Breastfed reference. ^2^ Data were modelled with a Parametric Growth Curve (PGC) mixed model with a quadratic function of time. The stratification factors of sex, site, and birth weight as covariate were added to the model. For the analyses including the Breastfed reference group, maternal BMI was included as an additional covariate for weight and length gain, and maternal education was also included as a covariate for length gain.

**Table 5:** Number and percentage of infants with any event and at least one adverse event in the system organ class of gastrointestinal disorder ^1^.

Adverse Event	Test (*n* = 107)	Control (*n* = 104)	*p*-Value ^3^	Breastfed (*n* = 61)
Any Event	42 (39.3)	33 (31.7)		15 (24.6)
Gastrointestinal disorders	22 (20.6)	17 (16.3)	0.431	6 (9.8)
Abdominal pain	2 (1.9)	3 (2,9)		0 (0.0)
Constipation	4 (3.7)	3 (2.9)	0.730	1 (1.6)
Diarrhoea	1 (0.9)	2 (1.9)		1 (1.6)
Flatulence	1 (0.9)	2 (1.9)		2 (3.3)
Infantile colic	8 (7.5)	4 (3.8)	0.256	1 (1.6)
Infantile vomiting	3 (2.8)	2 (1.9)		0 (0.0)
Regurgitation	6 (5.6)	1 (1.0)	0.060	1 (1.6)
Other ^2^	6 (5.6)	4 (3.8)		1 (1.6)

^1^ All subjects treated population and Breastfed reference, ^2^ Other gastrointestinal adverse events include for the Test group: abdominal distension (*n* = 1, 0.9%), dyschezia (*n* = 2, 1.9%), gastroesophageal reflux disease (*n* = 3, 2.8%); for the Control group: Abdominal pain upper (*n* = 1, 1.0%), anal fissure (*n* = 1, 1.0%), Dyschezia (*n* = 1, 1.0%), gastroesophageal reflux disease (*n* = 1, 1.0%); and for the BF group: haematochezia (*n* = 1, 1.6%), ^3^ *p*-value based on the Miettinen-Nurminen method, on subjects who did versus did not have one or more events, by study group (unadjusted *p*-value). *p*-values are generated only when the event incidence is at least 4 (in either group).

## Supplementary Materials

The following are available online at https://www.mdpi.com/2072-6643/12/11/3560/s1, Table S1: Absolute weight, length, and head circumference values from baseline to visit 5, Figure S1: Average Parent Reported Stool Frequency Among Infants in the Randomised Groups.
