# Supplementary material for: A Partly Fermented Infant Formula with Postbiotics Including 3′-GL, Specific Oligosaccharides, 2′-FL, and Milk Fat Supports Adequate Growth, Is Safe and Well-Tolerated in Healthy Term Infants: A Double-Blind, Randomised, Controlled, Multi-Country Trial
Source: Nutrients. 2020 Nov 20;12(11):3560. doi: 10.3390/nu12113560 (PMC7699816; doi:10.3390/nu12113560)
Supplement: Supplementary file 1 [file nutrients-12-03560-s001.pdf]

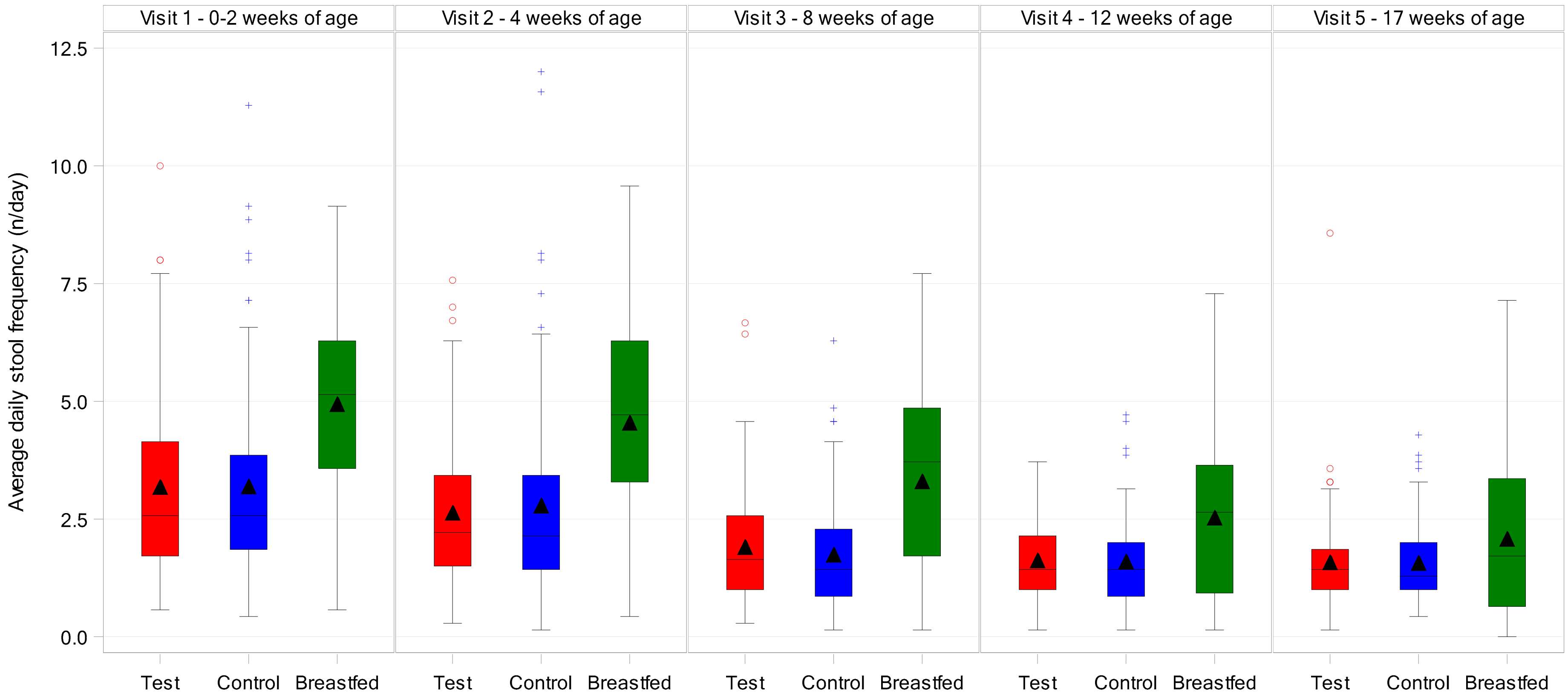

Figure S1. Average Parent Reported Stool Frequency Among Infants in the Randomised Groups[1, 2, 3.]

[1] All subjects treated population and Breastfed Reference

[2] Mann-Whitney test was used for comparison between the Test and Control groups (V1: $P = 0.893$; V2: $P = 0.708$; V3: $P = 0.372$; V4: $P = 0.690$; V5: $P = 0.986$)

[3] Number of subjects per group for Test, Control, and Breastfed group, respectively – V1: n = 103/99/59; V2: n = 100/97/59; V3: n = 94/89/59; V4: n = 90/87/56; V5: n = 90/87/56.

Table S1. Absolute weight, length, and head circumference values from baseline to visit 5[1].

	**Test**		Control		Breastfed	
	**Female**	**Male**	Female	Male	Female	Male
	Mean (SD)	Mean (SD)	Mean (SD)	Mean (SD)	Mean (SD)	Mean (SD)
Visit 1 (0-2 weeks of age)						
n	54	47	49	46	31	27
Weight	3313 (418)	3608 (439)	3322 (314)	3405 (396)	3400 (300)	3460 (375)
Length	51.6 (2.9)	52.8 (2.7)	51.9 (2.7)	52.3 (2.6)	51.3 (2.4)	52.3 (2.3)
Head Circumference	34.7 (1.1)	35.5 (1.0)	34.8 (0.8)	35.1 (1.0)	34.7 (0.9)	35.1 (1.1)
Visit 2 (4 weeks of age)						
n	52	45	47	42	28	27
Weight	4108 (457)	4482 (388)	4110 (371)	4254 (450)	4045 (353)	4252 (420)
Length	54.2 (2.4)	55.3 (2.1)	54.4 (2.3)	54.9 (2.5)	53.7 (2.3)	54.6 (2.5)
Head Circumference	36.5 (1.1)	37.4 (1.0)	36.4 (1.0)	36.7 (1.1)	36.1 (1.0)	36.9 (1.0)
Visit 3 (8 weeks of age)						
n	48	41	43	41	27	26
Weight	5023 (576)	5469 (427)	5011 (487)	5306 (569)	4849 (452)	5293 (518)
Length	57.2 (2.2)	58.4 (2.6)	56.9 (2.0)	58.0 (2.3)	56.5 (2.1)	57.8 (2.6)
Head Circumference	38.2 (1.1)	39.2 (1.0)	38.1 (0.8)	38.8 (1.1)	38.0 (0.9)	38.8 (1.2)
Visit 4 (12 weeks age)						
n	46	40	42	40	25	26
Weight	5910 (714)	6287 (469)	5810 (627)	6215 (607)	5566 (566)	6092 (572)
Length	59.9 (2.4)	61.4 (2.4)	59.5 (2.1)	60.9 (2.6)	59.5 (2.3)	60.6 (2.9)
Head Circumference	39.7 (1.2)	40.8 (1.0)	39.8 (0.9)	40.4 (1.2)	39.3 (0.7)	40.3 (1.2)
Visit 5 (17 weeks of age)						
n	45	40	42	39	25	25
Weight	6707 (839)	7119 (596)	6631 (697)	7065 (718)	6206 (703)	6890 (577)
Length	62.8 (2.3)	64.3 (2.2)	62.6 (1.9)	63.7 (2.6)	62.1 (2.5)	63.8 (2.9)
Head Circumference	41.2 (1.4)	42.4 (1.0)	41.4 (0.9)	41.8 (1.3)	40.7 (1.0)	41.9 (1.1)

[1] Per protocol population and Breastfed Reference.
